# Dual-Wavelength Fluorescence Polarization Immunoassay for Simultaneous Detection of Sulfonamides and Antibacterial Synergists in Milk

**DOI:** 10.3390/bios12111053

**Published:** 2022-11-21

**Authors:** Changfei Duan, Yingjie Zhang, Peipei Li, Qiang Li, Wenbo Yu, Kai Wen, Sergei A. Eremin, Jianzhong Shen, Xuezhi Yu, Zhanhui Wang

**Affiliations:** 1 Beijing Key Laboratory of Detection Technology for Animal-Derived Food, Beijing Laboratory for Food Quality and Safety, College of Veterinary Medicine, China Agricultural University, Beijing 100193, China; 2 Department of Chemistry, M.V. Lomonosov Moscow State University, 119991 Moscow, Russia

**Keywords:** sulfonamides, antibacterial synergists, dual-wavelength fluorescence polarization immunoassay, homogeneous detection

## Abstract

Combinations of sulfonamides (SAs) and antibacterial synergists (ASGs) are frequently used for treating infectious diseases and promoting growth for animals, which cause potential hazards to food safety and human health. To realize the simultaneous detection of SAs and ASGs in food, a homogeneous and high-throughput screening dual-wavelength fluorescence polarization immunoassay (DWFPIA) was developed. In this study, three SAs tracers and three ASGs tracers were synthesized by fluoresceins with different linkers and paired with their corresponding monoclonal antibodies (mAbs), respectively. To achieve a high sensitivity and broad specificity, the combination of tracers SADMPM-HDF with the longest linker paring mAb 10E6 for SAs and tracer HaptenA-DSCA paring mAb 9C9 for ASGs were chosen for the development of DWFPIA, achieving surprising IC_50_ values for 23 SAs below 100 μg L^−1^ and 5 ASGs below 50 μg L^−1^. The accuracy of DWFPIA was applied in real milk samples by typical sulfamethazine (SMZ) and trimethoprim (TMP), with recoveries of 81.7–97.2% and 78.6–103.6%, and coefficient of variations (CVs) below 18.9%, which could be completed within 15 min, including sample pretreatment. We firstly developed a simultaneous screening DWFPIA, covering all of the SAs and ASGs used in clinic and providing a great application potential in food safety analysis.

## 1. Introduction

Sulfonamides (SAs) are a class of synthetic antibacterials and have been widely employed to treat infectious diseases in human beings and animals, and they are also used as growth-promoting feed additives due to their advantages of having a high efficiency, low cost, excellent stability, large yield, and adequate supply [1,2]. Antibacterial synergists (ASGs), as the dihydrofolate-reductase inhibitor, are commonly utilized in combination with sulfonamides for enhancing the antibacterial activity [3,4]. However, animal-derived food may be contaminated by the residues of SAs and ASGs. In addition, the abuse, as well as illegal and long-term sub-therapeutic level usage of SAs and ASGs may cause the appearance of drug-resistant variants, which can cause a potential risk to human health and the entire ecosystem [1,5,6,7]. Because of these potential risks, the European Union and China enacted the maximum residue limits (MRLs) of SAs and ASGs in animal-derived foods; the MRLs for SAs are set at 100 μg kg^−1^ in the muscle, liver, kidney, and fat of food-producing animals, as well as in milk (except the MRLs for SMZ that is 25 μg kg^−1^ in the milk of in China) [8,9]. The MRLs for ASGs in the edible tissues of food-producing animals were about 30–50 μg kg^−1^ in the European Union, China, and other regulatory agencies [8,9,10]. Therefore, it is imperative and desirable to establish a rapid, sensitive, and selective method for the simultaneous monitoring of SAs and ASGs residues in food.

Numerous instrumental analytical methods have been utilized for the simultaneous detection of SAs and ASGs, such as gas chromatography and mass spectrometry, high-performance liquid chromatography, and liquid chromatography–tandem mass spectrometry [11,12,13,14,15]. There is a high degree of accuracy and sensitivity in the instrumental analysis methods; however, these methods require complex steps for sample preparation, expensive instruments, and skilled technicians, which are not very appropriate for the rapid detection of numerous samples in a short time. The potential applications of immunoassays, especially the enzyme-linked immunosorbent assay (ELISA), could be a solution to these problems regarding the screening of contaminants in food due to their low cost, high specificity, sensitivity, and without the need to rely on professional technicians. However, most traditional solid-phase immunoassays are still time-consuming because they also need to separate the free component from the antigen–antibody complex, and require multiple washing and incubation steps. Simplifying the operation procedure and minimizing the time are necessary for establishing rapid detection methods for high-throughput determination. Fluorescence polarization immunoassay (FPIA) is a homogeneous method without multiple separations or washing steps, which allows for a high-throughput, rapid, and quantitative analysis of the chemical contaminants within a few minutes. In the past few years, FPIA has been developed for monitoring veterinary drugs [16,17], environmental pollutants [18,19,20], pesticides [21,22,23], and toxins [24,25,26]. For more than a decade, our group has reported many FPIA methods to detect hazardous compounds, such as aflatoxins, zearalenone, sulfamethazine, orbifloxacin, and amantadine. [17,25,27,28,29]. However, to the best of our knowledge, no DWFPIA method has been reported for the simultaneous detection of SAs and ASGs in foodstuffs.

Because of the lack of a signal amplification process, the sensitivity of FPIA may be lower than ELISA [30]. To enhance the sensitivity of FPIA as much as possible, fluorescein-labeled derivatives (tracers) were carefully designed and synthesized. Li et al. synthesized three tracers with different linkers (2-, 4-, and 6-carbon) that were applied to test the influence on the tracer structures. The results showed that the fluoresceinthiocarbamyl hexamethylenediamine-labeled conjugate with the longer linker (6-carbon) had the best sensitivity in the FPIA for detection [22]. Chun et al. also reported three tracers containing different linkers (2-, 3-, and 6-carbon), and established a FPIA for the screening of zearalenone. These results demonstrated that the tracer that contained a 6-carbon linker obtained the most sensitive FPIA [31]. In this work, we synthesized dual-color tracers using different fluorescence dyes with different linker lengths and paired with specific monoclonal antibodies (mAbs) to perform the DWFPIA. After careful optimization, comparison, and analysis, a highly sensitive and specific DWFPIA was developed for the simultaneous detection of SAs and ASGs in milk.

## 2. Materials and Methods

### 2.1. Materials

Sulfamethazine (SMZ), sulfisomidine (SIM), sulfadimethoxine (SDM), sulfadoxine (SDM’), sulfamethoxydiazine (SMD), sulfamonomethoxine (SMM), sulfalene (SLE), sulfamethoxypyridazine (SMP), sulfaethoxypyridazine (SEP), sulfamerazine (SMR), sulfadiazine (SD), sulfabromomethazine (SBM), sulfachlorpyrazine (SCY), sulfachloropyridazine (SCP), sulfaquinoxaline (SQX), sulfapyridine (SPY), sulfabenzamide (SBA), sulfamoxole (SXL), sulfisoxazole (SIZ), sulfamethoxazole (SMX), sulfathiazole (STZ), sulfamethizole (SMT), and sulfaphenazole (SPA) were supplied by Millipore Sigma (St. Louis, MO, USA). Trimethoprim (TMP), diaveridine (DVD), and ormetoprim (OMP) were supplied from Dr. Ehrenstorfer GmbH (Ausburg, Germany). Brodimoprim (BOP) was supplied by J & K Chemical Technology (Beijing, China). Baquiloprim (BQP) was purchased from Sigma-Aldrich (St. Louis, MO, USA). Fluorescein derivatives, including fluoresceinthiocarbamyl ethylenediamine (EDF), fluoresceinthiocarbamyl butanediamine (BDF), and fluoresceinthiocarbamyl hexamethylenediamine (HDF), were synthesized in our study [17]. Disulfo-Cy5 amine (DSCA) and disulfo-Cy5 hydrazide (DSCH) were acquired from Confluore Biological Technology Co., Ltd. (Shanxi, China). Alexa Fluor 647 cadaverine was supplied by Thermo Fisher Scientific Inc. (Waltham, MA, USA). Haptens SADMPM and HaptenA were previously synthesized in our laboratory.

N,N-Dimethylformamide (DMF) was supplied by Aladdin (Shanghai, China). N-hydroxysuccinimide (NHS) and N,N′-dicyclohexylcarbodiimide (DCC) were supplied by Sigma-Aldrich (St. Louis, MO, USA). All of the other reagents were of analytical purity and were supplied by Sinopharm Chemical Reagent Co., Ltd. (Shanghai, China). Silica gel plates for thin layer chromatography (TLC) were supplied by Merck (Darmstadt, Germany). Low-adsorption opaque 96-well microplates were supplied by Corning (Oneonta, NY, USA). The anti-SAs mAbs 4C7 and 4D11 were previously reported by our group [32], and the mAb 10E6 is described elsewhere. In addition, the anti-ASGs mAbs 5C4, 9C9, 3B6, 14G1, and 1F1 were previously reported [3].

Borate buffer (BB, pH 8.0) was utilized as the diluent buffer for the DWFPIA experiments. The stock standards of SMZ and TMP (10 mg mL^−1^) were prepared and kept at −20 °C. The PerkinElmer microplate reader (EnVision2105, Waltham, MA, USA) was employed to record the FP signal.

### 2.2. Synthesis and Characterization of Tracers

In this study, the hapten of SADMPM was conjugated with EDF, BDF, and HDF to prepare the short-wavelength tracers (SAs group) for SAs detection (represented by SMZ); the hapten of HaptenA was conjugated with DSCA, DSCH, and AF647 to prepare the long-wavelength tracers (ASGs group) for ASGs detection (represented by TMP). All of the tracers were synthesized with the active ester method by the condensation of the amino group on fluorescein with the carboxyl group on SADMPM/HaptenA. Briefly, 3.5 mg of SADMPM/HaptenA was dissolved in 300 μL of DMF and mixed with NHS (2.3 mg) and DCC (3.3 mg), and then the mixtures were incubated for 3 h at room temperature with stirring. After a short period of centrifugation, 2.0 mg of fluorescein was dissolved in the supernatant and reacted overnight in the dark. To obtain the pure fluorescein tracer, the reaction solutions were purified by TLC using a trichloromethane/methanol/acetic acid mixture (6:1:0.07, *v*/*v*/*v*). The main band on TLC was collected and extracted with methanol. All of the the target tracers were characterized by FPIA using a specific mAb.

### 2.3. Development and Optimization of DWFPIA

#### 2.3.1. Protocol of the DWFPIA

For single format FPIA, 70 μL/well of a standard analyte solution, 70 μL/well of tracer, and 70 μL/well mAb were added to the 96-well low-adsorption microplate. After incubation for 2 min in the dark, FP values were measured at λ_ex_ 480 nm and λ_em_ 535 nm for the SAs tracers (SADMPM-EDF, SADMPM-BDF, and SADMPM-HDF) or at λ_ex_ 620 nm and λ_em_ 688 nm for the ASGs tracers (HaptenA-DSCA, HaptenA-DSCH, and HaptenA-AF647). For DWFPIA detection, 70 μL/well standard cocktail solutions were mixed with 70 μL/well of tracer cocktail solutions in the working dilution and 70 μL/well of mAb cocktail solutions in the working dilution. After incubation for 2 min in the dark, the corresponding signals of the tracers were collected simultaneously.

#### 2.3.2. Screening of Antibody-Tracer Pairs

In DWFPIA, we mainly focused on three analytical parameters to select the optimum antibody–tracer pairs, including the half-maximal inhibitory concentration (IC_50_), the detection window (δmP = mP_max_ − mP_min_), and the titer of the mAbs. To screen the best antibody–tracer pairs, δmP /IC_50_ was used as the primary parameter. The lower the IC_50_ of an antibody–tracer pair, the higher the sensitivity of the DWFPIA that developed. The IC_50_ of DWFPIA based on the antibody–tracer pairs was assessed according to standard curves, as described in Section 2.3.3. δmP was the difference between the observed maximum FP value of the tracer bound to a saturating amount of antibody (mP_max_) and the observed minimum FP value of the free tracer (mP_min_). In this work, the tracer with a suitable δmP (>80 mP) was employed for the following experiment. In addition, the binding abilities of the tracers and mAbs were assessed. Briefly, three anti-SAs mAbs and five anti-ASGs mAbs were performed with multiple dilutions using BB, separately, and reacted with two groups of different wavelength tracers, as described in Section 2.3.1. According to the antibody dilution curve, the titer of the mAbs utilized for each tracer that satisfied the δmP criterion was calculated.

#### 2.3.3. Competitive Standard Curves of DWFPIA

The procedure to develop the standard curves of DWFPIA for the simultaneous detection of SMZ and TMP was follows: the standard cocktail solutions were prepared by mixing an equal quantity of the standard of SMZ and TMP. Subsequently, the optimum two tracers for each mAbs were added with equal volumes, and then mixed with the two mAbs against SMZ and TMP. The other steps followed the above protocol for FPIA. The standard curves were constructed by plotting the FP values against the concentration of the corresponding standard SMZ and TMP; these data points were fitted with the four-parameter logistic function using OriginPro 8.0 (OriginLab Corp., Northampton, MA, USA).

Y = (A − D)/[1 + (X/C) ^B^] + D (1)

where Y represents the FP value for the corresponding different concentrations of the standard (analyte); A and D represent the asymptotic maximum and minimum of the FP values, respectively; and B is the slope of the curve at the inflection point. C is the IC_50_ value that defines the standard concentration of SMZ or TMP (analyte) at 50% tracer binding, and X is SMZ/TMP in various concentrations. Furthermore, IC_20_–IC_80_ was calculated as the detectable range of the DWFPIA. According to the following equation, the cross reactivity (CR) of the DWFPIA was acquired as follows:

CR= (IC_50_ of SMZ or TMP)/(IC_50_ of analyte compound) × 100% (2)

#### 2.3.4. Optimization of the DWFPIA

To obtain a sensitive FPIA, the effects of some physicochemical conditions on DWFPIA were evaluated by comparing the δmP/IC_50_ ratios under various conditions, including pH and organic solvents. Based on the optimal antibody–tracer pairs, pH values of 6.0, 7.0, 8.0, and 9.0 were tested and the other parameters of the assay remain unchanged. To clarify the effects of organic solvents on the development of DWFPIA, various concentrations of methanol and acetonitrile were tested, that is, 0%, 2.5%, 5%, 10%, 20%, and 30% (*v*/*v*) for methanol and 0%, 2.5%, 5%, 10%, and 20% (*v*/*v*) for acetonitrile. SMZ and TMP were used as the reference analyte in DWFPIA optimization to construct standard curves, seperately.

#### 2.3.5. Preparation of Milk Sample

The sample preparation was based on previous reports with minor modifications [33]. An amount of 2.5 mL of milk was mixed with an equal volume of 1.5% trichloroacetic acid (TCA) in 10 mL polypropylene centrifuge tubes, and vortexed for 1 min to precipitate the proteins, then the mixture was centrifuged for 5 min at 10,000× *g*. The supernatant was acquired and the pH was adjusted to 8.0 through the addition of 1.0 M NaOH solution. Finally, the supernatant was diluted a total of 10 times using the BB buffer and was analyzed using DWFPIA. The LOD of the DWFPIA was defined as the average value of 20 independent blank controls plus three times their standard deviation [34]. For the recovery experiments, blank milk was spiked with different amounts of the standard, respectively. After pretreatment, the samples were diluted and detected by DWFPIA. All of the recovery data were analyzed in triplicate (*n* = 3).

## 3. Results and Discussions

### 3.1. Preparation and Characterization of Tracers

**Figure 1:**
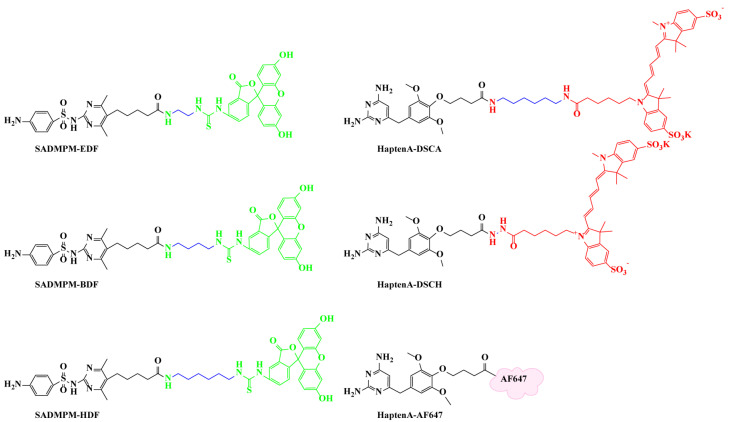
Tracers for SAs and ASGs with varied lengths of the linker are labelled in blue. The structures of short-wavelength tracers for SAs (**left** column) and long-wavelength tracers for ASGs (**right** column).

According to previous reports, the length of the linker between the hapten and fluorescein would directly affect the sensitivity of FPIA [35,36,37,38,39]. Generally, the linker between the fluorescein and hapten needs to separate the antibody binding site from the fluorescein molecule, so that the antibody can recognize the hapten of tracers through its inherent affinity, with as little steric hindrance as possible. In the meantime, the linker between the fluorescein and hapten can also reduce the quenching of fluorescein [40]. Therefore, various lengths of the linkers were designed. Three SAs tracers were conjugated by hapten SADMPM with different lengths of linker fluoresceins, EDF, BDF, and HDF, which increased in alkane linear length from 2 to 6 once by two carbon atoms, as shown in Figure 1. Three ASGs tracers were conjugated by haptenA with different lengths of linker fluoresceins (HaptenA-DSCA, HaptenA-DSCH, and HaptenA-AF647) (Figure 1). The fluorescein dye of DSCA, DSCH, and AF647 were bright red long-wavelength dye with excitation at 620 nm and emission at 688 nm. These are commonly combined with antibodies and proteins for cellular imaging [41,42,43], and have hardly any application for the detection of SAs and ASGs. In this work, the involvement of bright red long-wavelength dye in the DWFPIA was aimed to separate two signals in a single well, and to then obtain the goal of the simultaneous detection of SAs and ASGs. The tracers were separated by TLC, the main bands with R_f_ = 0.63 for SADMPM-EDF, R_f_ = 0.87 for SADMPM-BDF, R_f_ = 0.86 for SADMPM-HDF, R_f_ = 0.72 for HaptenA-DSCA, R_f_ = 0.63 for HaptenA-DSCH, and R_f_ = 0.44 for HaptenA-AF647 were collected (Figure S1 and Tables S1 and S2 in Supporting Information).

**Figure 2:**
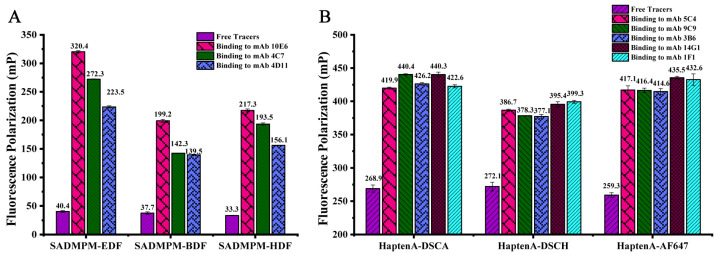
The tracers are characterized by the antibody binding assay. (**A**) The tracers of SAs had binding with 100-fold diluted mAbs of 10E6, 4C7, and 4D11 (*n* = 3). (**B**) The tracers of ASGs had binding with 100-fold diluted mAbs of 5C4, 9C9, 3B6, 14G1, and 1F1 (*n* = 3).

The six synthesized tracers were characterized by antibody–tracer binding assays. The FP signals of all of the tracers showed a significant increase before and after saturating, and the amounts of corresponding antibodies were added with δmP ranging from 101 mP to 280 mP for the SAs tracers and δmP ranging from 105 mP to 176 mP for the ASGs tracers (Figure 2). The results show that these tracers were synthesized and separated successfully.

### 3.2. Optimization of Antibody-Tracer Pairs

Different antibody–tracer pairs may have a crucial impact on the sensitivity and accuracy in the development of FPIA [29,35]. To obtain the best antibody–tracer pair, we calculated the antibody titers of each mAb–tracer pair according to the antibody dilution curves (Table 1 and Figure S2). In this work, the antibody titer is defined as the dilution value when there is 50% fixed tracer binding. For all of the SAs tracers, the titers of mAb 4C7 were too low (<1/1000), which indicated that mAb 4C7 and the SAs tracers were unsuitable for the following study. We then evaluated other mAb–tracer pairs with appropriate antibody titers (>1/1000; listed in Table 1) by separately constructing SMZ or TMP standard curves. The titer, IC_50_, δmP, and δmP/IC_50_ of the standard curves for all of the antibody–tracer pairs are summarized in Table 1. δmp/IC_50_ is the primary parameter to evaluate the sensitivity of the DWFPIA for each mAb–tracer pair, and higher values of δmp/IC_50_ mean a higher sensitivity for DWFPIA. As shown in Table 1, compared with tracers SADMPM-EDF and SADMPM-BDF with a shorter linker, the longer linker tracer SADMPM-HDF with mAb 10E6 produced the highest sensitivity, for which the IC_50_ and δmp/IC_50_ were 5.6 ng/mL and 18.8, respectively. In addition, this trend was also observed in ASGs tracers with mAbs, that is, the longer linker tracer HaptenA-DSCA with mAb 9C9 obtained the lowest IC_50_ of 1.0 ng/mL and the highest δmp/IC_50_ of 128.4. Thus, an increase in the linker length of the tracer significantly improved the sensitivity of the developed DWFPIA. Dong et al. employed a tracer containing the longest linker to minimize the fluorescence quenching of the fluorescein molecules and to reduce the interference at the antibody binding sites by the fluorescent tags paired with antibodies to develop the best performing FPIA [36]. There are two studies on the simultaneous detection of analytes using DWFPIA that have been reported [25,38]. However, the effect of linker length on sensitivity was not mentioned or clearly discussed. Our results further indicate that the longer linker between hapten and fluorescein was recommended to increase the sensitivity of DWFPIA. Thus, SADMPM-HDF-mAb 10E6 and HaptenA-DSCA-mAb 9C9 were the optimal antibody–tracer pairs and were selected to develop the DWFPIA for the detection of SAs and ASGs. At the beginning of the work, the tracer concentration was empirically used, which was nearly 10-fold higher than the background signal of BB. Thus, the working concentrations of the tracers were evaluated at FIs of 5, 10, and 20-fold greater than the FIs of the BB background, respectively. As shown in Figure S3A,B, the highest δmP/IC_50_ was selected when the concentrations of SADMPM-HDF and HaptenA-DSCA were 10-fold BB. The δmP/IC_50_ of the DWFPIA varied over time until the competitive recognition of the antibody, tracers, and analytes were at equilibrium. As shown in Figure S4A,B, the δmP/IC_50_ of the DWFPIA reached a plateau when the incubation time was at 2 min, thus this parameter was employed for the subsequent experiments.

### 3.3. Development of DWFPIA for SAs and ASGs

**Figure 3:**
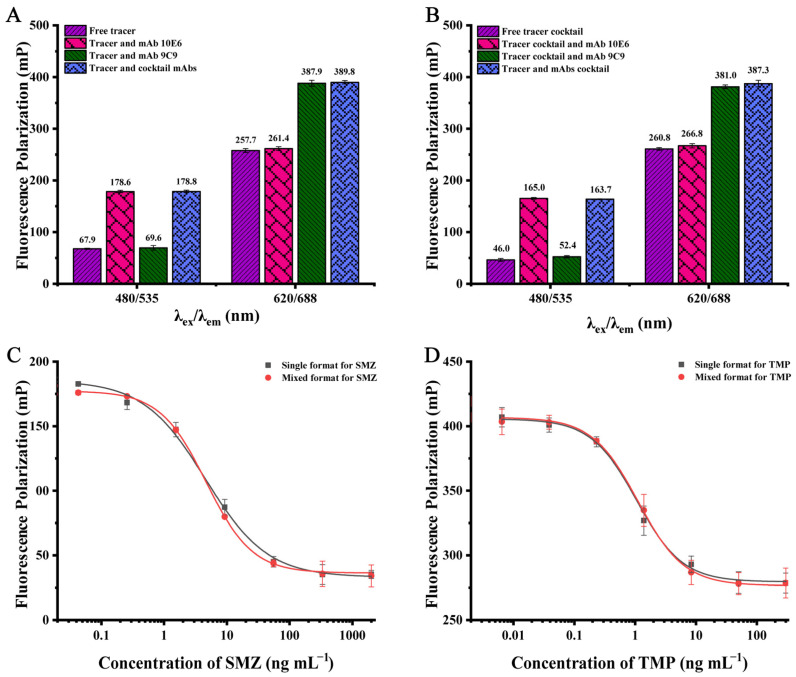
Binding of the single tracer (**A**) and the tracer cocktails (**B**) with mAbs at the working concentration. Standard curves for SMZ, TMP, and the standard cocktail in mixed format at different wavelengths: (**C**) λ_ex_ 480 nm/λ_em_ 535 nm (*n* = 3); (**D**) λ_ex_ 620 nm/λ_em_ 688 nm (*n* = 3).

The detection system of DWFPIA consists of two distinct fluorescence signals; moreover, the interference between the short-wavelength fluorescein (EDF/BDF/HDF) and the long-wavelength fluorescein (DSCA/DSCH/AF647) could be ignored, according to our previous studies [26,38]. Furthermore, the nonspecific binding was analyzed by comparing the FP changes of each tracer (or the tracer cocktail) upon the addition of the free, specific, non-specific, and cocktail mAbs at working concentrations. As shown in Figure 3A, the FP value of the free tracer SADMPM-HDF was 67.9 mP at 480 nm/535 nm, and the FP values had no significant difference with the addition of the non-specific mAb 9C9. Moreover, the FP values were significantly increased when the specific mAb 10E6 and cocktail mAbs of 10E6 and 9C9 were added with FP values of 178.6 mP and 178.8 mP. The results showed that the increased FP values of SADMPM-HDF were mainly caused by the specific mAb 10E6 and were not affected by the non-specific mAb 9C9. At 620 nm/688 nm, the binding of the tracer HaptenA-DSCA and its corresponding antibody mAb 9C9 was not affected by the presence of non-specific mAb 10E6, as shown in Figure 3A. Similar results were also observed in the cocktail tracers, as shown in Figure 3B. The results indicate that the mixture of two tracers and two antibodies did not have non-specific binding with each other.

In DWFPIA, the change in FP values at a given λex/λem should be primarily induced by the corresponding target analyte. Therefore, the inhibition test of DWFPIA was detected at different λex/λem by contrasting the decrease in FP values when the analyte was added in a single or mixed format. As shown in Figure 3C, the SMZ primarily competed with the corresponding tracer in the cocktail solution at 480 nm/535 nm, as the shapes of the SMZ standard curves in the single and mixed formats were nearly identical and the IC_50_ values were similar (4.55 ng mL^−1^ and 4.67 ng mL^−1^). In addition, at 620 nm/688 nm, TMP mainly inhibited the binding of HaptenA-DSCA to the corresponding mAb 9C9, as the shapes of the TMP standard curves in the single and mixed formats were almost coincidental and the IC_50_ was 1.06 ng mL^−1^ and 1.12 ng mL^−1^ (Figure 3D). The comparative results of the binding and inhibition studies revealed that the mixture of two tracers (SADMPM-HDF and HaptenA-DSCA) and two mAbs (10E6 and 9C9) did not appear to have non-specific binding with each other. Thus, the tracers and mAbs mixture could be utilized to develop DWFPIA for simultaneously detecting the residue of SMZ and TMP in food.

### 3.4. Optimization of the DWFPIA for SAs and ASGs

**Figure 4:**
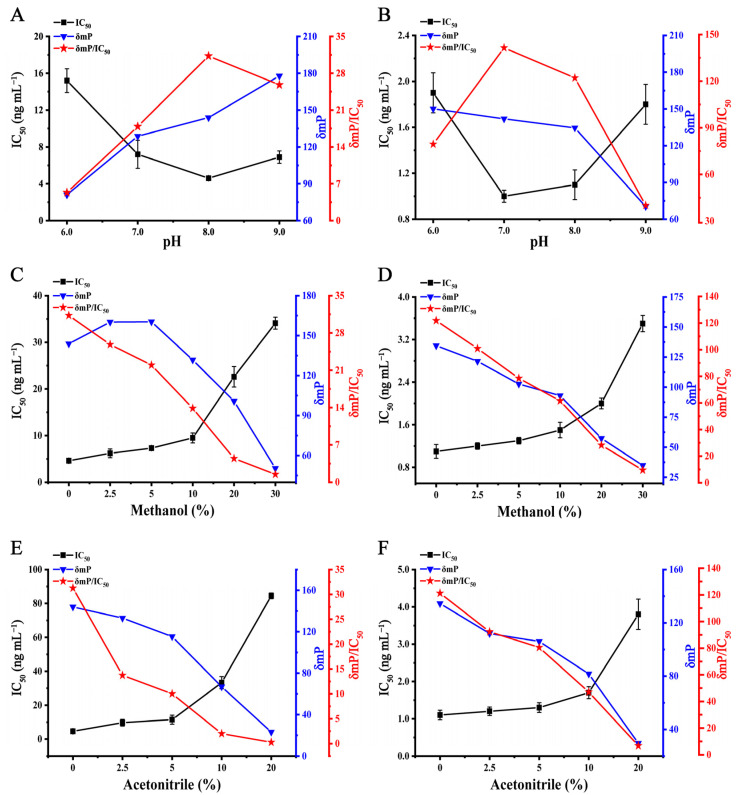
Influence of pH in BB on DWFPIA for SMZ (**A**) and TMP (**B**). Methanol tolerance of DWFPIA for SMZ (**C**) and TMP (**D**). Acetonitrile tolerance of DWFPIA for SMZ (**E**) and TMP (**F**).

In the developed DWFPIA, fluoresceins were pH-sensitive materials, and the recognition of the tracer and antibodies could be obviously changed by the pH value of the detection system [39,44]. To obtain a high sensitivity of assays, the effect of pH on the DWFPIA was assessed. The analyte (SMZ/TMP), tracer, and mAb cocktails were mixed in a BB solution with a pH of 6.0, 7.0, 8.0, and 9.0. δmP/IC_50_ was employed as the principal criteria for screening of the suitable condition. As shown in Figure 4A, the highest δmP/IC_50_ was obtained at pH 8.0 for SMZ with δmP/IC_50_ of 31.3, while the highest δmP/IC_50_ was presented at pH 7.0 for TMP with δmP/IC_50_ of 141.9, with a slight difference in δmP/IC_50_ of pH 8.0 (Figure 4B). Thus, pH 8.0 was chosen as the optimal pH for the next studies.

Organic solvents are commonly used in the pretreatment of food samples, and a suitable concentration of organic solvents could increase the solubility of the standards and tracers. On the other hand, excessive organic solvents may cause a denaturation of antibodies and a lower sensitivity of assays [3,45,46]. Methanol and acetonitrile are the most commonly used organic solvents for pretreatment, so we investigated the methanol and acetonitrile tolerance by mixing the analyte cocktail, tracer, and mAb cocktails, diluted in BB (pH 8.0, containing different concentration of methanol/acetonitrile). With the methanol concentration being increased from 0 to 30%, the δmP/IC_50_ of the DWFPIA decreased for the detection of SMZ and TMP (Figure 4C,D). δmP/IC_50_ decreased significantly at 20–30% methanol and the IC_50_ rose dramatically, revealing that the final methanol concentration of the assay system was no more than 10%. As seen in Figure 4E,F, acetonitrile had a greater impact on the overall system, δmP/IC_50_ changed significantly with the acetonitrile concentration having increased, and δmP < 80 mP for SMZ at a 10% final acetonitrile concentration (Figure 4E). However, there was little affect with a final concentration of 10% acetonitrile for the detection of TMP (Figure 4F), because the IC_50_ changed slightly from 1.1 to 1.7 ng mL^−1^ and the δmPs were greater than 80 mP. The results revealed that the sensitivity of the detection system had no significant changes when the final concentration of acetonitrile was 5%.

### 3.5. Characteristics of DWFPIA and their Application in Milk

**Figure 5:**
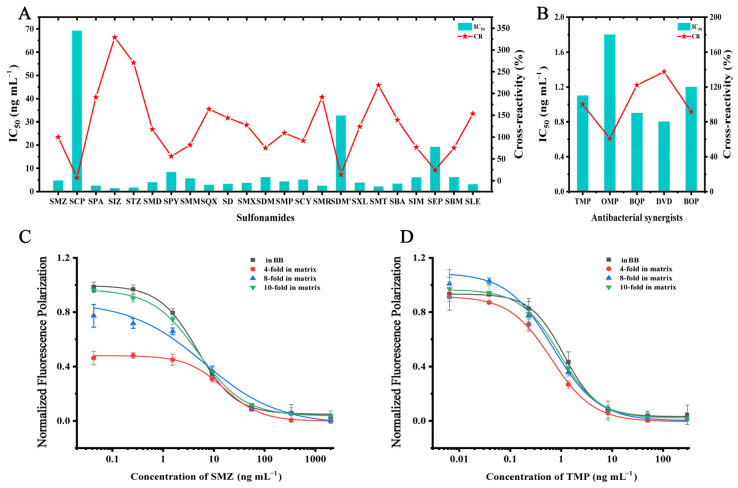
The IC_50_ values and cross-reactivity (CR) of the developed DWFPIA to selected SAs (**A**) and ASGs (**B**). (**C**) Normalized standard curves of SMZ in a BB buffer and milk with different dilutions. (**D**) Normalized standard curves of TMP in a BB buffer and milk with different dilutions.

SAs and ASGs are commonly utilized in combination in the veterinary clinic, so it is crucial that the developed DWFPIA can be used for simultaneous detection. Based on the optimized conditions, the recognizable spectrum of DWFPIA was determined by CRs with 23 SAs and 5 ASGs. As shown in Figure 5A,B and Tables S3 and S4, the results showed that the established DWFPIA had an excellent identification of SAs with CRs from 6.8% to 328.6% and ASGs with CRs from 61.1% to 137.5%, demonstrating that DWFPIA could detect at least 23 SAs and 5 ASGs as set by the European Union and China.

The homogeneous immunoassay is susceptible to interference by the matrix effect caused by different components present in the samples. Removing the matrix effect is necessary to ensure the accuracy and precision of the immunoassay. Milk is a complex system containing different fats, proteins, and sugars, which may hinder the specific recognition between the antibody and antigen [47]. Generally, there are two methods to eliminate the matrix effect, including solid-phase extraction (SPE) and dilution. The SPE is time-consuming and expensive, and requires intensive labor, which makes it unsuitable for the rapid screening method. In this study, the SMZ and TMP standard curve was prepared in BB and compared with the standard curves prepared by the extracts after 4, 8, and 10 dilutions. The data were processed and normalized according to a previous study [48]. As shown in Figure 5C,D, the influence of the matrix on the detective performance of the DWFPIA reduced as the dilution increased. The shape of the normalized calibration curve in 10-times dilution of the extract was almost the same with standard curves in BB, indicating that the matrix effects were almost eliminated by simple sample pretreatment (as described in Section 2.3.5). The LOD for SMZ was 3.3 μg L^−1^, the detectable range for SMZ was 10.7–221.9 μg L^−1^, while the LOD for TMP was 0.7 μg L^−1^ and the detectable range for TMP was 2.2–37.7 μg L^−1^ (Table 2). There have been instrumental analytical methods introduced in papers reporting on the simultaneous detection of SAs and ASGs. For comparison, their main results are summarized in Table S5 [11,12,49,50], in the Supporting Information. The present DWFPIA was shown to be sensitive enough to detect these two types of drug residues and had a much shorter assay time (2 min) than those methods, which is a crucial characteristic for the rapid and simple detecting assay.

Moreover, the accuracy and precision of the optimized DWFPIA were determined by spiked milk samples, and the recoveries and coefficient of variation (CV) were evaluated. Blank milk was spiked with a standard cocktail of SMZ (20, 50, 100 μg L^−1^) and TMP (5, 20, 35 μg L^−1^) at three different concentrations. As shown in Table 2, the recoveries were 81.7–97.2% for SMZ and 78.6–103.6% for TMP, with the corresponding CV ranging from 8.0–18.9% and 7.8–17.1%, respectively, which demonstrated that the developed DWFPIA had potential utility for detecting SAs and ASGs in milk samples.

## 4. Conclusions

In this study, we first applied the haptens of SAs and ASGs to synthesize the three SAs tracers and three ASGs tracers with different linkers between the hapten and fluorescein, and paired with their corresponding mAbs for the analysis of DWFPIA. The results showed that the SADMPM-HDF and HaptenA-DSCA tracers containing the longer linker were perfect when paired with corresponding mAb 10E6 and 9C9 for the development of the DWFPIA, respectively. Moreover, we tested the potential nonspecific binding between the optimal tracer–mAb pairs of SAs and ASGs. The results demonstrated that there was no nonspecific binding between each other, which was the foundation for the development of the DWFPIA. Subsequently, DWFPIA was developed for screening SAs and ASGs simultaneously, and the LOD, dynamic range, and specificity were evaluated. Finally, the accuracy and precision of the DWFPIA in the milk was tested by recovery assays.

## Figures and Tables

**Table 1:** Characteristics of each tracer–antibody pair in the BB buffer.

Tracer-mAb Pairs	Parameters			
	Titers	IC_50_ (ng mL^−1^)	δmP	δmP/IC_50_
SADMPM-EDF-10E6	1/2110	17.6	101.9	5.8
SADMPM-BDF-10E6	1/1620	15.2	98.7	6.5
SADMPM-HDF-10E6	1/2027	5.6	105.5	18.8
SADMPM-EDF-4C7	1/649	- ^a^	- ^a^	- ^a^
SADMPM-BDF-4C7	1/384	- ^a^	- ^a^	- ^a^
SADMPM-HDF-4C7	1/443	- ^a^	- ^a^	- ^a^
SADMPM-EDF-4D11	1/4381	44.2	84.1	1.9
SADMPM-BDF-4D11	1/2764	38.4	72.2	1.9
SADMPM-HDF-4D11	1/6301	36.0	71.9	2.0
HaptenA-DSCA-5C4	1/3270	1.8	117.9	65.5
HaptenA-DSCH-5C4	1/5007	2.5	102.2	40.9
HaptenA-AF647-5C4	1/4751	2.3	129.2	56.2
HaptenA-DSCA-9C9	1/3485	1.0	128.4	128.4
HaptenA-DSCH-9C9	1/4929	1.4	99.7	71.2
HaptenA-AF647-9C9	1/6160	1.1	123.9	112.6
HaptenA-DSCA-3B6	1/3879	3.0	127.8	42.6
HaptenA-DSCH-3B6	1/6491	3.8	99.9	26.3
HaptenA-AF647-3B6	1/4223	2.2	100.1	45.5
HaptenA-DSCA-14G1	1/4591	1.8	156.1	86.7
HaptenA-DSCH-14G1	1/6037	2.2	133.5	60.7
HaptenA-AF647-14G1	1/5883	1.5	150.7	100.5
HaptenA-DSCA-1F1	1/8093	3.8	150.0	39.5
HaptenA-DSCH-1F1	1/8066	5.0	121.2	24.2
HaptenA-AF647-1F1	1/12,785	3.0	153.4	51.1

^a^ Not calculated.

**Table 2:** Analytical characteristics of DWFPIA for the detection of SMZ and TMP, for the recoveries and CVs of spiked blank samples (*n* = 3).

Analytes	Parameters (μg L^−1^)				
	LOD	Detectable Range	Spiked Concentration	Recovery (%)	CV (%)
SMZ	3.3	10.7–221.9	20	81.7	18.9
			50	97.2	12.6
			100	84.5	8.0
TMP	0.7	2.2–37.7	5	78.6	13.4
			20	103.6	17.1
			35	90.5	7.8

## Data Availability

The data presented in this study are available upon request from the corresponding author.

## Supplementary Materials

The following supporting information can be downloaded at: https://www.mdpi.com/article/10.3390/bios12111053/s1. Figure S1: The TLC purification of the tracers. The target band is enclosed in a red box. Figure S2: Antibody dilution curves of tracers (*n* = 3). (A) mAb 10E6 with SADMPM-EDF, SADMPM-BDF, and SADMPM-HDF. (B) mAb 4C7 with SADMPM-EDF, SADMPM-BDF, and SADMPM-HDF. (C) mAb 4D11 with SADMPM-EDF, SADMPM-BDF, and SADMPM-HDF. (D) mAb 5C4 with HaptenA-DSCA, HaptenA-DSCH, and HaptenA-AF647. (E) mAb 9C9 with HaptenA-DSCA, HaptenA-DSCH, and HaptenA-AF647. (F) mAb 3B6 with HaptenA-DSCA, HaptenA-DSCH, and HaptenA-AF647. (G) mAb 14G1 with HaptenA-DSCA, HaptenA-DSCH, and HaptenA-AF647. (H) mAb 1F1 with HaptenA-DSCA, HaptenA-DSCH, and HaptenA-AF647. Table S1: R_f_ values of the short-wavelength tracers of SAs. Table S2: R_f_ values the long-wavelength tracers of ASGs. Table S3: The IC_50_ values and CRs in DWFPIA of SAs. Table S4: The IC_50_ values and CRs in DWFPIA of ASGs. Table S5: Comparisons with reported simultaneous analytical methods for SAs and ASGs.

## References

[1] Zhu N., Zhu Y., Wang J., Gyimah E., Hu X., Zhang Z. (2019). A novel fluorescence immunoassay based on AgNCs and ALP for ultrasensitive detection of sulfamethazine (SMZ) in environmental and biological samples. Talanta.

[2] Liang X., Li C., Zhu J., Song X., Yu W., Zhang J., Zhang S., Shen J., Wang Z. (2019). Dihydropteroate synthase based sensor for screening multi-sulfonamides residue and its comparison with broad-specific antibody based immunoassay by molecular modeling analysis. Anal. Chim. Acta.

[3] Li H., Ma S., Zhang X., Li C., Dong B., Mujtaba M.G., Wei Y., Liang X., Yu X., Wen K. (2018). Generic Hapten Synthesis, Broad-Specificity Monoclonal Antibodies Preparation, and Ultrasensitive ELISA for Five Antibacterial Synergists in Chicken and Milk. J. Agric. Food Chem..

[4] Choi M.J., Yohannes S.B., Lee S.J., Damte D., Reza M.A., Rhee M.H., Kim T.H., Park S.C. (2012). The in vitro antibacterial activity of enrofloxacin-trimethoprim combination against five bacterial species. Pak. Vet. J..

[5] Zamora-Gálvez A., Ait-Lahcen A., Mercante L.A., Morales-Narváez E., Amine A., Merkoçi A. (2016). Molecularly Imprinted Polymer-Decorated Magnetite Nanoparticles for Selective Sulfonamide Detection. Anal. Chem..

[6] Livermore D.M., Mushtaq S., Warner M., Woodford N. (2014). Comparative in vitro activity of sulfametrole/trimethoprim and sulfamethoxazole/trimethoprim and other agents against multiresistant Gram-negative bacteria. J. Antimicrob. Chemother..

[7] Lombardo M.N., G-Dayanandan N., Wright D.L., Anderson A.C. (2016). Crystal Structures of Trimethoprim-Resistant DfrA1 Rationalize Potent Inhibition by Propargyl-Linked Antifolates. ACS Infect. Dis..

[8] The European Union (2010). Commission Regulation (EU) No 37/2010 of 22 December 2009 on Pharmacologically Active Substances and Their Classification Regarding Maximum Residue Limits in Foodstuffs of Animal Origin.

[9] The Ministry of Agriculture and Rural Affairs of the People’s Republic of China (2019). National Food Safety Standard-Maximum Residue Limits for Veterinary Drugs in Foods.

[10] The Department of Food Safety, Ministry of Health, Labour and Welfare (2006). No. 0526004 of June 2006 for Agricultural Chemical Residues in Foods.

[11] Sichilongo K., Mutsimhu C., Obuseng V.C. (2013). Gas chromatography—Mass spectral characteristics of six pharmacologically active compounds—Analytical performance characteristics on a raw sewage impacted water sample. Can. J. Chem..

[12] Xu Z.-G., DU Z., Hu Y.-L., Pan Y.-P., Li G.-K. (2012). Preparation of Trimethoprim Molecularly Imprinted Stir Bar Sorptive Extraction and Its Application for Trace Analysis of Trimethoprim and Sulfonamides in Complex Samples. Chin. J. Anal. Chem..

[13] Chen D., Yu J., Tao Y., Pan Y., Xie S., Huang L., Peng D., Wang X., Wang Y., Liu Z. (2016). Qualitative screening of veterinary anti-microbial agents in tissues, milk, and eggs of food-producing animals using liquid chromatography coupled with tandem mass spectrometry. J. Chromatogr. B Anal. Technol. Biomed. Life Sci..

[14] Gavilán R.E., Nebot C., Patyra E., Miranda J.M., Franco C.M., Cepeda A. (2018). Simultaneous analysis of coccidiostats and sulphonamides in non-target feed by HPLC-MS/MS and validation following the Commission Decision 2002/657/EC. Food Addit. Contam. Part A Chem. Anal. Control Exposure Risk Assess.

[15] Croubels S., Wassink P., De Backer P. (2002). Simultaneous determination of sulfadiazine and trimethoprim in animal feed by liquid chromatography with UV and tandem mass spectrometric detection. Anal. Chim. Acta.

[16] Zhang S., Wang Z., Nesterenko I.S., Eremin S.A., Shen J. (2007). Fluorescence polarisation immunoassay based on a monoclonal antibody for the detection of sulphamethazine in chicken muscle. Int. J. Food Sci. Technol..

[17] Mi T., Liang X., Ding L., Zhang S., Eremin S.A., Beier R.C., Shen J., Wang Z. (2014). Development and optimization of a fluorescence polarization immunoassay for orbifloxacin in milk. Anal. Methods.

[18] Wang K., Liu Z., Ji P., Liu J., Eremin S.A., Li Q.X., Li J., Xu T. (2016). A camelid VHH-based fluorescence polarization immunoassay for the detection of tetrabromobisphenol A in water. Anal. Methods.

[19] Raysyan A., Moerer R., Coesfeld B., Eremin S.A., Schneider R.J. (2021). Fluorescence polarization immunoassay for the determination of diclofenac in wastewater. Anal. Bioanal. Chem..

[20] Liu L.-H., Zhou X.-H., Xu W.-Q., Song B.-D., Shi H.-C. (2014). Highly sensitive detection of sulfadimidine in water and dairy products by means of an evanescent wave optical biosensor. RSC Adv..

[21] Wang Y., Li Z., Barnych B., Huo J., Wan D., Vasylieva N., Xu J., Li P., Liu B., Zhang C. (2019). Investigation of the Small Size of Nanobodies for a Sensitive Fluorescence Polarization Immunoassay for Small Molecules: 3-Phenoxybenzoic Acid, an Exposure Biomarker of Pyrethroid Insecticides as a Model. J. Agric. Food Chem..

[22] Li M., Liu X., Hua X., Yin W., Fang Q., Wang M. (2014). Fluorescence polarization immunoassay for highly efficient detection of clothianidin in agricultural samples. Anal. Methods.

[23] Xu Z.-L., Wang Q., Lei H.-T., Eremin S.A., Shen Y.-D., Wang H., Beier R.C., Yang J.-Y., Maksimova K.A., Sun Y.-M. (2011). A simple, rapid and high-throughput fluorescence polarization immunoassay for simultaneous detection of organophosphorus pesticides in vegetable and environmental water samples. Anal. Chim. Acta.

[24] Lippolis V., Porricelli A.C.R., Mancini E., Ciasca B., Lattanzio V.M.T., De Girolamo A., Maragos C.M., McCormick S., Li P., Logrieco A.F. (2019). Fluorescence Polarization Immunoassay for the Determination of T-2 and HT-2 Toxins and Their Glucosides in Wheat. Toxins.

[25] Zhang X., Tang Q., Mi T., Zhao S., Wen K., Guo L., Mi J., Zhang S., Shi W., Shen J. (2018). Dual-wavelength fluorescence polarization immunoassay to increase information content per screen: Applications for simultaneous detection of total aflatoxins and family zearalenones in maize. Food Control.

[26] Li C., Wen K., Mi T., Zhang X., Zhang H., Zhang S., Shen J., Wang Z. (2016). A universal multi-wavelength fluorescence polarization immunoassay for multiplexed detection of mycotoxins in maize. Biosens. Bioelectron..

[27] Wang Z.-H., Zhang S.-X., Shen J.-Z., Sergei A.E. (2007). Analysis of Sulfamethazine by Fluorescence Polarization Immunoassay. Chin. J. Anal. Chem..

[28] Chen M., Wen K., Tao X., Ding S., Xie J., Yu X., Li J., Xia X., Wang Y., Xie S. (2014). A novel multiplexed fluorescence polarisation immunoassay based on a recombinant bi-specific single-chain diabody for simultaneous detection of fluoroquinolones and sulfonamides in milk. Food Addit. Contam. Part A Chem. Anal. Control Exposure Risk Assess.

[29] Guo L., Liu M., Li Q., Dong B., Li H., Mari G.M., Liu R., Yu W., Yu X., Wang Z. (2021). Synthesis and characterization of tracers and development of a fluorescence polarization immunoassay for amantadine with high sensitivity in chicken. J. Food Sci..

[30] Oberleitner L., Grandke J., Mallwitz F., Resch-Genger U., Garbe L.-A., Schneider R.J. (2014). Fluorescence Polarization Immunoassays for the Quantification of Caffeine in Beverages. J. Agric. Food Chem..

[31] Chun H.S., Choi E.H., Chang H.-J., Choi S.-W., Eremin S.A. (2009). A fluorescence polarization immunoassay for the detection of zearalenone in corn. Anal. Chim. Acta.

[32] Wang Z., Beier R.C., Sheng Y., Zhang S., Jiang W., Wang Z., Wang J., Shen J. (2013). Monoclonal antibodies with group specificity toward sulfonamides: Selection of hapten and antibody selectivity. Anal. Bioanal. Chem..

[33] Liang X., Sheng Y., Yu W., Zhao S., Shan H., Zhang Q., Wang Z. (2018). Comparison of Chicken IgY and Mammalian IgG in Three Immunoassays for Detection of Sulfamethazine in Milk. Food Anal. Methods.

[34] Wang X., Wu X., Lu Z., Tao X. (2020). Comparative Study of Time-Resolved Fluorescent Nanobeads, Quantum Dot Nanobeads and Quantum Dots as Labels in Fluorescence Immunochromatography for Detection of Aflatoxin B1 in Grains. Biomolecules.

[35] Chen Y., He Q., Shen D., Jiang Z., Eremin S.A., Zhao S. (2019). Fluorescence polarization immunoassay based on a new monoclonal antibody for the detection of the Diisobutyl phthalate in Yoghurt. Food Control.

[36] Dong B., Zhao S., Li H., Wen K., Ke Y., Shen J., Zhang S., Shi W., Wang Z. (2019). Design, synthesis and characterization of tracers and development of a fluorescence polarization immunoassay for the rapid detection of ractopamine in pork. Food Chem..

[37] Lippolis V., Pascale M., Valenzano S., Pluchinotta V., Baumgartner S., Krska R., Visconti A. (2011). A rapid fluorescence polarization immunoassay for the determination of T-2 and HT-2 toxins in wheat. Anal. Bioanal. Chem..

[38] Guo L., Liu M., Zhang S., Wang Z., Yu X. (2021). Multi-wavelength fluorescence polarization immunoassays for simultaneous detection of amantadine and ribavirin in chicken and human serum. Food Agric. Immunol..

[39] Yang J.-Y., Zhang Y., Wang H., Xu Z.-L., Eremin S.A., Shen Y.-D., Wu Q., Lei H.-T., Sun Y.-M. (2014). Development of fluorescence polarisation immunoassay for carbofuran in food and environmental water samples. Food Agric. Immunol..

[40] Wang Q., Haughey S.A., Sun Y.-M., Eremin S.A., Li Z.-F., Liu H., Xu Z.-L., Shen Y.-D., Lei H.-T. (2011). Development of a fluorescence polarization immunoassay for the detection of melamine in milk and milk powder. Anal. Bioanal. Chem..

[41] Chen S., Wang J., Xin B., Yang Y., Ma Y., Zhou Y., Yuan L.-J., Huang Z.-L., Yuan Q. (2019). Direct Observation of Nanoparticles within Cells at Subcellular Levels by Super-Resolution Fluorescence Imaging. Anal. Chem..

[42] Xu W., Su P., Zheng L., Fan H., Wang Y., Liu Y., Lin Y., Zhi F. (2018). In vivo Imaging of a Novel Strain of Bacteroides fragilis via Metabolic Labeling. Front. Microbiol..

[43] Subbiah N., Campagna J., Spilman P., Alam M.P., Sharma S., Hokugo A., Nishimura I., John V. (2017). Deformable Nanovesicles Synthesized through an Adaptable Microfluidic Platform for Enhanced Localized Transdermal Drug Delivery. J. Drug Deliv..

[44] Mi T., Wang Z., Eremin S.A., Shen J., Zhang S. (2013). Simultaneous Determination of Multiple (Fluoro)quinolone Antibiotics in Food Samples by a One-Step Fluorescence Polarization Immunoassay. J. Agric. Food Chem..

[45] Lei H., Xue G., Yu C., Haughey S.A., Eremin S.A., Sun Y., Wang Z., Xu Z., Wang H., Shen Y. (2011). Fluorescence polarization as a tool for the detection of a widely used herbicide, butachlor, in polluted waters. Anal. Methods.

[46] Cháfer-Pericás C., Maquieira Á., Puchades R., Miralles J., Moreno A. (2010). Fast screening immunoassay of sulfonamides in commercial fish samples. Anal. Bioanal. Chem..

[47] Zvereva E.A., Zherdev A., Formanovsky A.A., Abuknesha R.A., Eremin S.A., Dzantiev B.B. (2018). Fluorescence polarization immunoassay of colchicine. J. Pharm. Biomed. Anal..

[48] Maragos C.M., Jolley M.E., Plattner R.D., Nasir M.S. (2001). Fluorescence Polarization as a Means for Determination of Fumonisins in Maize. J. Agric. Food Chem..

[49] Cheng S., Wei Z., Zhiming X., Yang L., Xia F. (2021). Trace analysis and identification of 33 sulfonamides and sulfonamide potentiators in eggs by ultrahigh-performance liquid chromatography coupled with quadrupole-high-field orbitrap high-resolution mass spectrometry. Anal. Methods.

[50] Babić S., Ašperger D., Mutavdžić D., Horvat A.J., Kaštelan-Macan M. (2006). Solid phase extraction and HPLC determination of veterinary pharmaceuticals in wastewater. Talanta.

