# Supplementary material for: Dual-Wavelength Fluorescence Polarization Immunoassay for Simultaneous Detection of Sulfonamides and Antibacterial Synergists in Milk
Source: Biosensors (Basel). 2022 Nov 21;12(11):1053. doi: 10.3390/bios12111053 (PMC9688798; doi:10.3390/bios12111053)
Supplement: Supplementary file 1 [file biosensors-12-01053-s001.zip › biosensors-2018847-supplementary.pdf]

Supporting information for

Dual-wavelength fluorescence polarization immunoassay for simultaneous detection of sulfonamides and antibacterial synergists in milk

Changfei Duan [1], Yingjie Zhang [1], Peipei Li [1], Qiang Li [1], Wenbo Yu [1], Kai Wen [1], Sergei A. Eremin [2], Jianzhong Shen [1], Xuezhi Yu [1,*] and Zhanhui Wang [1]

[1] *Beijing Key Laboratory of Detection Technology for Animal-Derived Food, Beijing Laboratory for Food Quality and Safety, College of Veterinary Medicine, China Agricultural University, 100193 Beijing, People's Republic of China*

[2] *Department of Chemistry, M.V. Lomonosov Moscow State University, Moscow 119991, Russia*

**Corresponding author:* Xuezhi Yu

E-mail: yu51422396@126.com

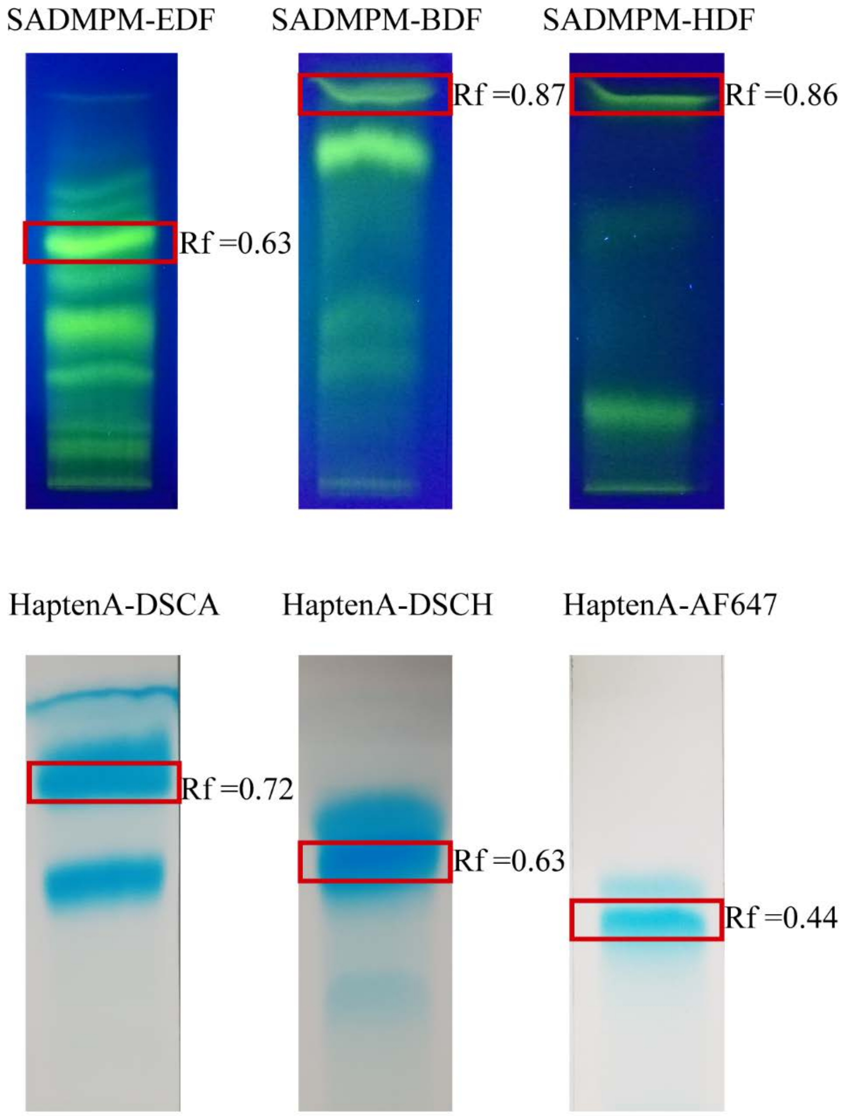

Figure S1. The TLC purification of the tracers. The target band is enclosed in a red box.

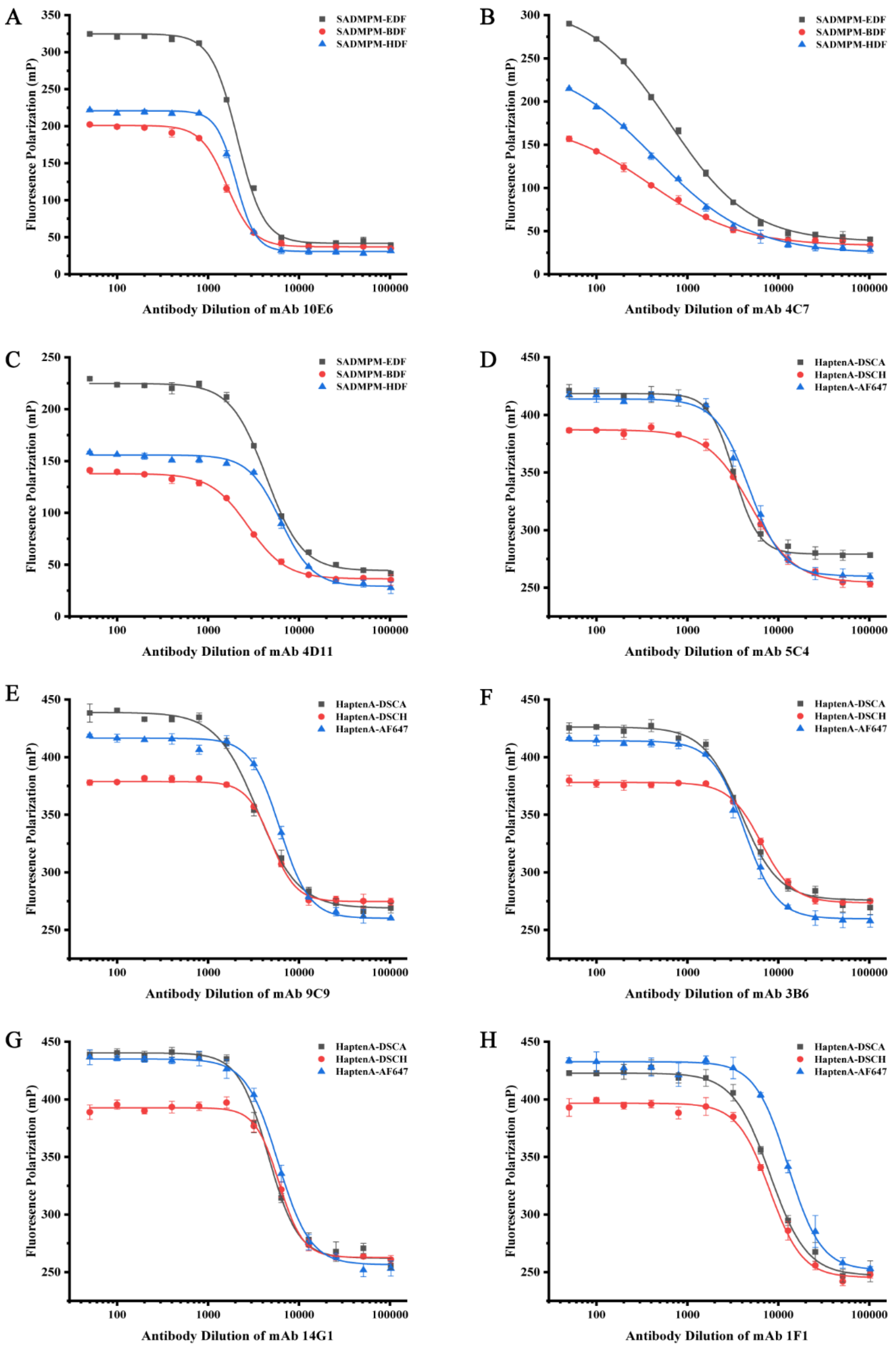

Figure S2. Antibody dilution curves of tracers (n = 3). (A) mAb 10E6 with SADMPM-EDF, SADMPM-BDF, and SADMPM-HDF. (B) mAb 4C7 with SADMPM-EDF, SADMPM-BDF, and SADMPM-HDF. (C) mAb 4D11 with

SADMPM-EDF, SADMPM-BDF, and SADMPM-HDF. (D) mAb 5C4 with HaptenA-DSCA, HaptenA-DSCH, and HaptenA-AF647. (E) mAb 9C9 with HaptenA-DSCA, HaptenA-DSCH, and HaptenA-AF647. (F) mAb 3B6 with HaptenA-DSCA, HaptenA-DSCH, and HaptenA-AF647. (G) mAb 14G1 with HaptenA-DSCA, HaptenA-DSCH, and HaptenA-AF647. (H) mAb 1F1 with HaptenA-DSCA, HaptenA-DSCH, and HaptenA-AF647.

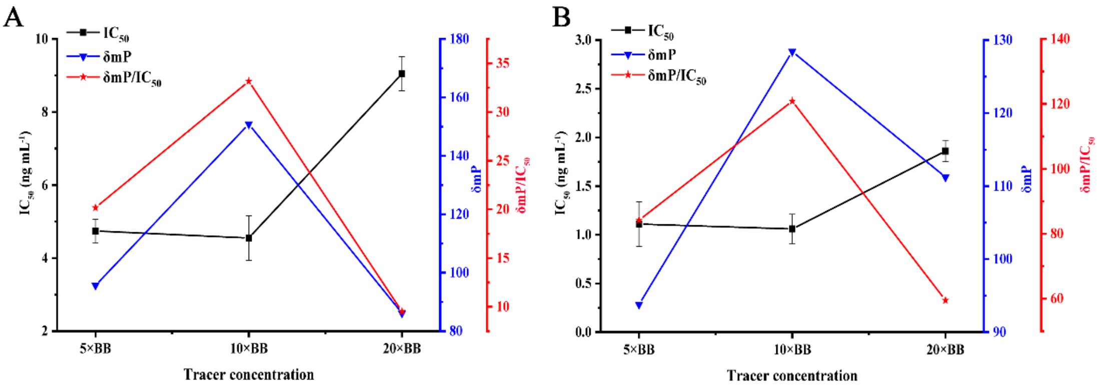

Figure S3. The effect of the tracer concentration for SMZ (A) and TMP (B).

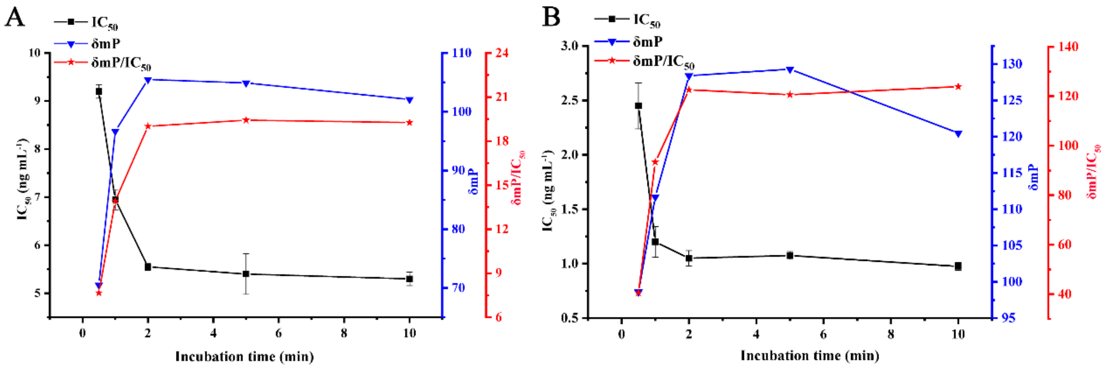

Figure S4. Influence of incubation time on DWFPIA for SMZ (A) and TMP (B)

Table S1. R_f values of the short-wavelength tracers of SAs.

Names	Volume (μl)	Developing solvent	R_f values
SADMPM-EDF	20	trichloromethane/methanol/acetic acid (6:1:0.07, v/v/v)	0.63
SADMPM-BDF	20	trichloromethane/methanol/ acetic acid (6:1:0.07, v/v/v)	0.87
SADMPM-HDF	20	trichloromethane/methanol/ acetic acid (6:1:0.07, v/v/v)	0.86

Table S2. R_f values the long-wavelength tracers of ASGs.

Names	Volume(μl)	Developing solvent	R_f values
HaptenA-DSCA	20	trichloromethane/methanol/ acetic acid (6:1:0.07, v/v/v)	0.72
HaptenA-DSCH	20	trichloromethane/methanol/ acetic acid (6:1:0.07, v/v/v)	0.63
HaptenA-AF647	20	trichloromethane/methanol/ acetic acid (6:1:0.07, v/v/v)	0.44

Table S3. The IC_{50} values and CRs in DWFPIA of SAs

Name	IC_{50}（ng/mL）	CRs（%）
SMZ	4.7	100
SCP	69.2	6.8
SPA	2.5	191.7
SIZ	1.4	328.6
STZ	1.7	270.6
SMD	4.0	117.9
SPY	8.4	56.1
SMM	5.7	82.1
SQX	2.9	164.3
SDZ/SD	3.3	143.8
SMX	3.7	127.8
SDM	6.2	75.4
SMP	4.3	109.5
SCY	5.1	92.0
SMR	2.5	191.7
SDM′	32.6	14.4
SXL	3.8	124.3
SMT	2.1	219.0
SBA	3.4	139.4
SIM	6.1	76.7
SEP	19.2	24.5
SBM	6.2	75.4
SLE	3.1	153.3

Table S4. The IC_{50} values and CRs in DWFPIA of ASGs

Name	IC_{50}（ng/mL）	CRs（%）
TMP	1.1	100
OMP	1.8	61.1
BQP	0.9	122.2
DVD	0.8	137.5
BOP	1.2	91.7

Table S5. Comparisons with reported simultaneous analytical methods for SAs and ASGs.

methods	analytes number	assay time (from added samples)	LOD ($\mu g\ L^{-1}$ or $\mu g\ kg^{-1}$)	references
GC-MS	1 SAs<br>1 ASGs	—[a]	SMZ 1140[b]<br>TMP 860[b]	[11]
MIP-SBSE-HPLC	3 SAs<br>1 ASGs	—[a]	SMZ 4.8<br>TMP 1.6	[12]
UHPLC-QE HF HRMS	27SAs<br>6ASGs	10 min	SMZ 0.01<br>TMP 0.02	[49]
SPE-HPLC	3 SAs<br>1 ASGs	20 min	SMZ 10<br>TMP 10	[50]
DWFPIA	23 SAs<br>5 ASGs	2 min	SMZ 3.3<br>TMP 0.7	this study

[a] No report

[b] positive chemical ionization (selected ion monitoring) method detection limits

Gas chromatography-mass spectral (GC-MS)

Molecularly imprinted polymer-coated stir bar sorptive extraction coupled with high performance liquid chromatography (MIP-SBSE-HPLC)

Ultra-high performance liquid chromatography coupled to quadrupole-high-field orbitrap high resolution MS (UHPLC-QE HF HRMS)

Solid phase extraction high-performance liquid chromatography (SPE-HPLC)
